# Approximate entropy: a promising tool to understand the hidden electrical activity of fruit

**DOI:** 10.1080/19420889.2023.2195236

**Published:** 2023-03-28

**Authors:** Gabriela Niemeyer Reissig, Thiago Francisco de Carvalho Oliveira, André Geremia Parise, Ádrya Vanessa Lira Costa, Douglas Antônio Posso, Cesar Valmor Rombaldi, Gustavo Maia Souza

**Affiliations:** a Laboratory of Plant Cognition and Electrophysiology, Department of Botany, Institute of Biology, Federal University of Pelotas, Pelotas, Brazil; b School of Biological Sciences, University of Reading, Reading, UK; c Department of Agroindustrial Science and Technology, Faculty of Agronomy Eliseu Maciel, Federal University of Pelotas, Pelotas, Brazil

**Keywords:** Electrome, fruit herbivory, fruit ripening, machine learning, plant electrophysiology

## Abstract

Fruits, like other parts of the plant, appear to have a rich electrical activity that may contain information. Here, we present data showing differences in the electrome complexity of tomato fruits through ripening and discuss possible physiological processes involved. The complexity of the signals, measured through approximate entropy, varied along the fruit ripening process. When analyzing the fruits individually, a decrease in entropy values was observed when they entered the breaker stage, followed by a tendency to increase again when they entered the light red stage. Consequently, the data obtained showed a decrease in signal complexity in the breaker stage, probably due to some physiological process that ends up predominating to the detriment of others. This result may be linked to processes involved in ripening, such as climacteric. Electrophysiological studies in the reproductive stage of the plant are still scarce, and research in this direction is of paramount importance to understand whether the electrical signals observed can transmit information from reproductive structures to other modules of plants. This work opens the possibility of studying the relationship between the electrical activity and fruit ripening through the analysis of approximate entropy. More studies are necessary to understand whether there is a correlation or a cause-response relationship in the phenomena involved. There is a myriad of possibilities for the applicability of this knowledge to different areas, from understanding the cognitive processes of plants to achieving more accurate and sustainable agriculture.

Plants have a rich and intense electrical activity. The first report of electrical signals in plants was provided by Sir John Burdon-Sanderson, through the measurement of the voltage difference between the adaxial and abaxial leaf surface of the Venus flytrap (*Dionaea muscipula* Ellis), while stimulating the hairs of the other half of the trap [1]. Initially, it was believed that only sensitive plants could have this property since bioelectrical phenomena were mostly associated with the movement of animals. Later on, at the onset of the 20^th^ century, Sir Jagadish Chandra Bose’s electrophysiological studies showed the existence of action potentials (APs) and intense, uninterrupted, and oscillatory electrical activity in ordinary plants, with no apparent rapid movements [2]. Nowadays, it is known that these signals influence several physiological processes, such as photosynthesis (e.g., variation potential inducing photosynthetic response, see [3], phytohormone responses (e.g., coupled to production and/or action of jasmonate or ethylene in wounding, see [4], and also plant responses to different environmental stimuli, being involved in local and long-distance signaling (e.g., rapid long-distance electrical signal associated with reactive oxygen species and Ca^2+^ waves, see [5].

One of the first consequences of environmental stimuli on plants is alteration in the plants’ electrical activity. Electrical signals, along with hydraulic signals, may participate in the induction of early systemic physiological responses to local stressors due to its fast propagation [6]. Based on this, recent studies are using electrophysiological data to monitor plants and make early diagnoses of stimuli/stresses of both abiotic and biotic origin [7,8,9,15]. Usually, these studies consider the whole plant or structures such as leaves or stems.

But what do we know about electrical signaling in reproductive structures, especially fruits? Would it be advantageous for plants to have a pathway for transmitting information originated in the fruit, despite the fruit being a structure ‘meant’ to be eaten? [10] found one of the first documented evidences of electrical signals between fruit and the aerial part of the plant. They observed the transmission of electrical signals between zucchini fruit (*Cucurbita pepo* L. var. *medullosa* Alef.) and the petiole of the underlying mature leaf, where changes in membrane potentials of the sieve tube elements at one location were accompanied by a change of potential on the other part only few seconds after the application of a sucrose solution.

Recently, our research group has provided evidence that supports [10], study. We found classifiable differences in the electrome of tomato fruits during ripening [43], as well as systemic electrical signaling from fruits being chewed by caterpillars toward the rest of the aerial part of the plant (Reissig et al., 2021a). These changes in the electrome are likely associated with physiological and cognitive processes [11–13]. Broadly, the plant electrome is an epiphenomenon based on the general definition given by [44] as the totality of ionic currents of any living entity, from the cell up to whole-organism level. Thus, plant electrome corresponds to the plants’ bioelectrical activity measured as micro-voltage changes in stimulated or non-stimulated tissues [14].

We have previously published works demonstrating that signal complexity analyses, such as approximate entropy (ApEn), provided important features for Machine Learning (ML) classification of the fruit electrome changes (Reissig et al., 2021a, 43). Now, we bring some unpublished data, obtained from the experiment conducted by [15], that is part of larger effort to understand these signals in fruits. Here, we have focused on ApEn, which was a parameter that stood out in many of our previous studies.

## Complexity, entropy, and fruit ripening

Plants are complex dynamical systems that do not possess a centralized processing unit. Instead, plants are built of several units/modules that interact with each other through self-organized processes, which leads to the emergence of what is observed as plant responses and behavior [16]. To achieve this, there must be communication between all the modules that compose a plant to secure the integration and coordination of the plant as a whole. This communication is deployed through a series of mechanisms such as hormones, RNAs, hydraulic cues, calcium signaling, and electrical signals. Quite often, more than one of these signaling pathways interact to sustain plant ecophysiological processes [5,17].

Plant cells can generate electrical signals when perceiving stimuli. There are many different electrical signals produced by plant cells, like action potentials, systems potentials and variation potentials [18,19]. Usually, these signals are initiated by cell membrane depolarization through the influx of calcium ions (Ca^2+^) to the cytoplasm. These signals run from cell to cell through plasmodesmata, sometimes propelled by chemical signals, and also can travel long distances through the vascular bundles, which ensures long-distance electrical signaling [18,20,21]. All these electrical signals operating at different scales and timeframes, when superimposed, result in an overall plant electrical activity, the plant electrome [14]. Since it emerges from the activity of underlying bioelectrical processes, no matter at which organ or part of the plant, the electrome can be observed at the level of individual plant organs, such as leaves, stem, inflorescences, roots, or even fruits.

The electrome is studied through the recording and analysis of electrophysiological time series, and there are different techniques that can be used to understand its dynamics. Among the analyses used to assess the level of complexity of electrophysiological time series [22], ApEn gives suitable and reliable information about the complexity of the signals, based on likely patterns found in the time series [23,24]. In short, low ApEn values indicate a low level of complexity, and their patterns tend to be repetitive and predictable. High ApEn values indicate a higher level of complexity, and the patterns are more irregular (complex) and difficult to predict. Thus, we can infer the amount of information (*sensu* [25] that the electrome carries. Furthermore, ApEn differs from other entropy analyses because it can discern changes in complexity from relatively small and noisy amounts of data [23].

Briefly, the calculation of Approximate Entropy, *ApEn (m,r)*, follows the equation [26]

$$ApEn\left(m,r\right)=\varphi^{m}\left(r\right)-\varphi^{m+1}\left(r\right)$$

where $\varphi^{m}=\frac{1}{N-m+1}\overset{N-m+1}{\underset{i=1}{\sum }}ln\left(d_{m,r}\left(i\right)/\left(N-m+1\right)\right)$ and $d_{m,r}\left(i\right)$ is the number (*i*) of vectors pairs m-dimensional to an Euclidian distance less or equal to *r*, and *N-m + 1* is the total number of vectors in the embedding dimension *m*. For this study, according to the demonstrations of Pincus [26] and [45], we assume *r = 0.2* (20% of the standard deviation of the time series ΔV) and *m* = 2.

For instance, in the study by Reissig et al. (2021a), ApEn was the feature that stood out the most, enabling accuracy of 90% in the classification by ML of electrome time series before and after herbivory in the fruit. Interestingly, a sharp difference in the electrome of the green and ripe fruit’s peduncles before herbivory, but not during it, was also observed. The signals generated by herbivory were predominant, making them easily classifiable by ML. This finding was a clue for us to start trying to understand the role of electrical signals in physiological processes such as fruit maturation where until then no relationship had been made. Thus, in [43], when monitoring the electrome of ripening tomato fruits detached from the plant, ApEn also appeared as the main feature that differentiated maturation stages through ML tools, demonstrating the importance of this trait for the characterization of fruit electrome.

**Figure 1.**
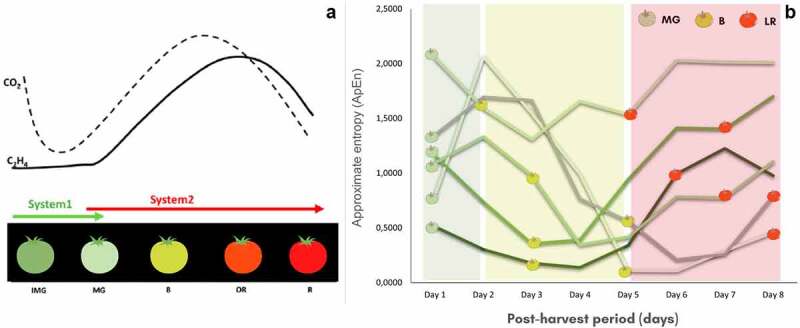
Scheme representing fruit ripening and its different ethylene biosynthesis systems (A, adapted from Liu et al., 2015) and the entropy measures (B). Each line in B represents a repetition of fruit analyzed up to the or stage. CO_2_: carbon dioxide; C_2_H_4_: ethylene; IMG: immature green stage; MG: mature green stage; B: breaker stage; OR: orange red stage; LR: light red stage; A: red stage.

When we analyzed the ApEn data separately, we noticed a pattern in each fruit as it ripened (Figure 1A). When the fruits reached the breaker stage, through daily visual monitoring, a decrease in ApEn values was observed. When the fruit began to transition to the light red stage, there was a tendency for the ApEn to rise again. What could be behind such changes in the dynamics of the fruit’s electrome complexity during ripening? Considering the fruit model studied and the ApEn pattern found, climacteric of Micro-Tom tomatoes and ethylene biosynthesis appear as prominent candidates to explain these results. This result suggests a relationship, but it is not enough to establish the co-variation or cause-effect responses. Studies inhibiting synthesis (pTOM 13) or blocking ethylene action by 1-MCP application, could contribute to answer this question. In Figure 1, we drawn a scheme to show the behavior of ethylene and climacteric synthesis (Figure 1A) and the ApEn pattern observed in six replicates throughout the ripening period. Climacteric behavior, present in fruits such as tomatoes, is characterized by an increase in respiratory rate accompanied by a peak in ethylene production [46]. Ethylene production can occur in two ways during fruit ripening. In the breaker stage, the transition from ethylene synthesis system 1 to system 2, which is more intense and autocatalytic, occurs.

Ethylene can activate calcium-permeable plasma membrane channels and is related to increases in cytoplasmic Ca^2+^ in plant cells [27,28]. It is proposed that ethylene causes the influx of Ca^2+^ by two pathways: directly, by activating ethylene receptors that stimulate Ca^2+^ channels, and indirectly, by inducing a burst of extracellular H_2_O_2_ that not only activate Ca^2+^ channels but also enhance the translation of these proteins [28]. The influx of Ca^2+^ is associated with a plethora of physiological processes such as regulating gene expression, wall ingrowth during papillae formation, and root hair and pollen tube elongation [27–29]. Besides, Ca^2+^ influx influences plant electrome since Ca^2+^ is the main ion involved in electrical signaling and its influx alters the membrane potential of plant cells [14]. Therefore, ethylene could be at least partly responsible for the electrophysiological alterations observed in tomato ripening.

Despite being a plant organ that performs little or no photosynthesis, the fruit, through the mitochondria, can drive a flow of electrons, generating energy and reactive oxygen species [30,31]. This flow of electrons is part of plants’ vital electrical activity and occurs through the electron transport chain at the membranes of chloroplasts and mitochondria. Briefly, the chain of the photochemical phase of photosynthesis is composed of a set of proteins that transport electrons from the water molecule to the ferredoxin-NADPH reductase; while in respiration, from the NADH or NADPH toward oxygen, generating water [14]. Currently, several works have already shown that electrical signals originating from external disturbances influence photosynthesis (e.g., fast systemic photosynthetic response connected with long-distance electrical signaling, see [32], but little is known about whether the electrical activity generated by photosynthesis and respiration could carry some information about internal processes, especially in reproductive modules.

**Figure 2.**
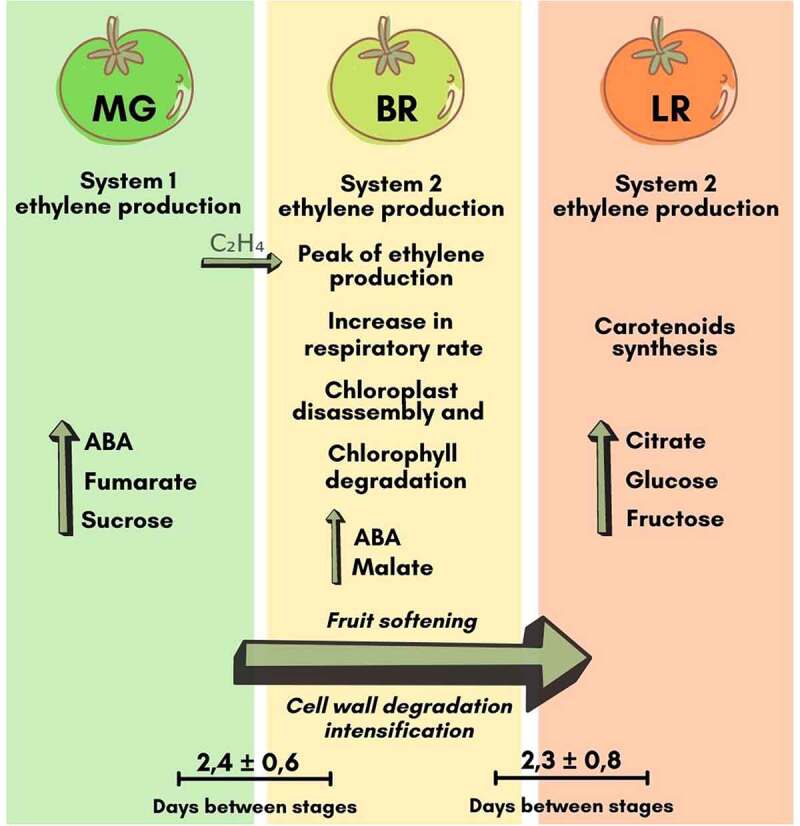
Summary of physiological and biochemical processes during fruit ripening [33–36]. The average and standard deviation of the days between stages were obtained in our experiment. C_2_H_4_: Ethylene; ABA: Abscisic acid.

In addition to ethylene synthesis and respiration, other processes and biochemical alterations might lead to electrical signatures specific to each ripening stage, due to the movement of ions, electrons, and protons in the cells and tissues [14]. In the mature green stage, the climacteric peak has not yet occurred (Figure 1A). The breaker and light red stages show more similarities, such as the increment in respiration rate and autocatalytic ethylene synthesis (Liu et al., 2015). Moreover, chlorophyll degradation, carotenoids accumulation, and cell wall lysis are other examples of common processes between both these stages (Figure 2). Once more, it is important to emphasize that the study was conducted with a climacteric fruit. More investigations are still needed on non-climacteric fruits, fruits not detached from the plant and dry fruits, to understand the dynamics of the electrical signal and its complexity throughout ripening in different kinds of fruits.

**Figure 3.**
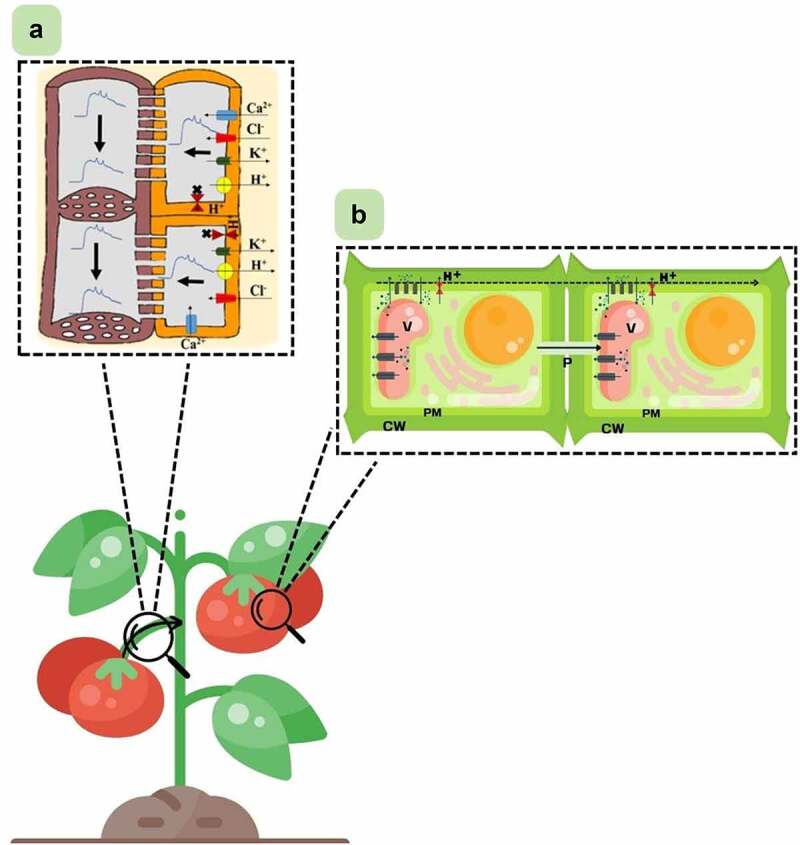
Scheme representing possible ways of propagating electrical signals from fruit to plant and between cells within the fruit. A: Representation of an action potential generated and transported along phloem cells (adapted from [14]. The curved black arrow on the petiole represents the propagation direction of the fruit-plant electrical signal. The brown structures represent the sieve tube elements, and the orange ones represent the companion cells. The small colored shapes represent ion channels and proton pumps. Thin black arrows on the membrane represent the influx and efflux of ions. Thick black arrows represent the direction of signal passage through phloem cells. B: Representation of cell-to-cell electrical signal transmission within the fruit (adapted from [5]. Components in gray represent ion channels, and those in red represent proton pumps. The small black and blue dots represent ions. The blue arrow represents the influx and efflux of ions and protons. The black dashed arrow demonstrates the direction of propagation of the signal across the membrane, and the solid black arrow through the plasmodesmata. V: vacuole; P: plasmodesmata; PM: plasma membrane; CW: cell wall; H+: proton.

Concerning the propagation of electrical signals in fruit, possibly, it is like what is observed in other parts of the plant. To simplify what is already known about the propagation of signals in plants, we made a scheme representing, in general, how this could occur in the fruit (Figure 3) without detailing its components, considering that they vary according to the type of signal and the stimulus studied. Phloem cells are one of the main routes of electrical signal transmission. They have relatively wide sizes and have large pore plates in the connection between them. Those cells also suffer partial apoptosis that devoid them of vacuole, nucleus, and plastids. All these properties make the sieve tubes of phloem highly suitable for the conductivity of electrical signals. In the studies by Reissig et al. (2021a), after predation of the fruit by a caterpillar, it was possible to observe changes in the electrome of the plant in the peduncle that connects the fruit to the plant, demonstrating the passage of a signal there. Possibly, this propagation could be via vascular bundles through the phloem, regardless of the mass flow (Figure 3A).

Within the fruit, signal propagation can occur from cell to cell via plasma membrane, cell wall, and plasmodesmata (Figure 3B) [5,37]. In general, the signals are generated and propagated by ionic flows through cell membranes crossed by ionic channels and ionic pumps. Such structures are highly diverse, where different stimuli trigger different channels and pumps, generating different signals [14,38]. Here, it is important to emphasize that the electrome is the totality of ionic currents of any living entity, from the cell up to the whole organism. It considers several signals at the same time, not individual ones, showing the entire analyzed tissue, not just a single cell, being the result of “n” intra and extracellular interactions. Within the plant, cells will interact, and depending on this interaction, new dynamics will emerge. In this sense, the electrome, unlike individual signals from the cells, does not linearly transmit information since the interaction of the cells occurs chaotically due to their multi-interaction behavior and multiple inputs that feed on each other. Due to this chaotic characteristic, it is difficult to define a specific transmission route. Thus, we analyze the final product of these interactions: the electrome.

Our next steps are to discover ways to correlate the electrophysiological data and the ApEn of the signals obtained with the physiological processes occurring at each ripening stage. However, one limitation is finding ways to perform analyzes that do not impact fruit dynamics and consider the underlying individuality of each ApEn pattern in the analyzed fruit. As can be seen in Figure 1B, although it is possible to visualize a general pattern, each fruit presented different transition times and entropy values. Damineli et al. [39] highlighted that biological oscillations such as electrical activity can be hard to predict, representing an epiphenomenon of a plant phenomenon, rather than presenting some specific biological function. The authors also mention that oscillatory processes are manifestations of underlying processes and that emergent characteristics of oscillatory processes, such as frequency and amplitude, can become important properties for information transmission. In this sense, the search for analyzes in well-conducted studies that demonstrate the relationship between electrical signals and plant processes are crucial for the advancement of plant electrophysiology.

The data obtained is interesting for basic science questions about electrical signals and cognitive processes in plants, and for application from a purely technological point of view. There are studies demonstrating that fruits can perceive environmental stimuli and warn the rest of the plant through electrical signals, independently of the mass flow of the vascular system [43]. In this same study, it was possible to verify the possibility of capturing electrome differences in the petiole/peduncle without damaging the fruit. Similarly, for the plant as a whole, it is possible to use the electrome for early diagnosis of diseases [8] and nutritional deficiencies [9]. Regarding the nutritional aspect of fruits and accumulation of sugars, the influx and efflux of molecules and ions in the cell leaves “electrical signatures” that can probably be identified through electrome analysis. There is still no work on this, specifically in the fruit. Nonetheless, studies have already been carried out on the plant, demonstrating the possibility of identifying nutritional deficiencies and differences [7,40]. The following steps are to find techniques to analyze the fruits and discover the electrome patterns behind the levels of sugars and nutrients, together with ML techniques. The potential applicability of these techniques to agriculture is evident.

Electrophysiology focused on reproductive structures is a field that still needs to be explored and presents numerous possibilities. The study of electrical signals coupled with the analysis of complex systems [41] and ML techniques are increasingly proving to be important tools in the most diverse areas of study. One example is applying fruit classification through electrome analysis coupled with ML techniques to refine the harvest of different fruits. Some fruits, such as those with a green color when ripe, present difficulties in automating and mechanizing harvesting due to their similarity with the immature fruit and the environment [42]. It is conjectured that ML’s multidimensional view could provide better results. The use of different features beyond images is an option to add more information layers to improve ML results. Our previous results have shown that electrophysiological analysis of plants can be used for this purpose [43]. For example, ML could be trained for relating pictures of the fruit ripening stages to the electrome at each point. This first process can create a much more precise classification of ripening, possibly far beyond the stages described in the literature. In addition, plant electrophysiology has enormous potential for increasingly accurate and sustainable food production and contributes greatly to the cultivation and post-harvest of fruits, as well as the understanding of the processes that occur in them.

## Material and methods

Seeds of cherry tomato (*Solanum lycopersicum* var. cerasiforme) were germinated in a polystyrene honeycomb germination box filled with a commercial organic substrate (kept moist by spraying distilled water daily), where they remained for 7 days in a germinating chamber (25°C, photoperiod of 12 h). After this period, the seedlings were transplanted to 1.0 L plastic pots filled with commercial organic substrate. The tomato plants were grown in a greenhouse at the Capão do Leão campus of the Federal University of Pelotas (31° 52′ 32′′ S and 52° 21′ 24′′ W, altitude 13 m). The average temperature in the greenhouse during the experimental period was 28.5 ± 12.9°C and the irradiance, from natural light, was on average 800 µmol photons m^−2^ s^−1^.

After transplantation, the plants were watered on alternate days (100 mL) and supplied with 50 mL of Hoagland and Arnon’s nutrient solution three times a week. When the fruits were green and fully established (before progressing to the breaker stage) they were harvested randomly from 50 tomato plants and transferred to laboratory conditions (25.0 ± 2.0°C, photoperiod of 12 h) where the experiment was carried on. In each essay, 1 day before the signal recording, four fruits were placed in a Faraday’s cage and a pair of needle electrodes (EL452 model; Biopac Systems, Goleta, CA, EUA) was inserted where the vascular bundles were visible. The electrodes of each fruit were 1 cm apart from each other. Five repetitions with four fruits each were made, totaling 20 fruit samples. The fruit ripening stages were categorized as follows: mature green (MG), breaker (BR) and light red (LR). We recorded the fruits’ electrome while they transitioned through all the ripening stages, from MG to LR. The bioelectrical signals were acquired during the fruit ripening in data acquisitions throughout 24 h until all fruits were in the LR stage.

Electrical signals were recorded with the electronic system for data acquisition MP36 (Biopac Systems, Goleta, CA, EUA), composed of four channels with high input impedance (10 GΩ). The signals were acquired by fixing a sampling rate of fs = 62.5 Hz with two filters, one high-pass (0.5 Hz cutoff frequency) and the other low-pass (1.5 kHz cutoff frequency). The bioelectrical runs were analyzed as voltage variation (µV) time series ΔV = {Δ*V*_1_, Δ*V*_2_, …, Δ*V*_N_} in which Δ*V*_i_ is the difference of potential between the inserted electrodes, scored in each $\frac{1}{f_{s}}$ time interval, and *N* is the total length of the series. The procedures for calculating ApEn were based on the original work of Pincus [26].
